# High-precision pothole detection using the ECC-YOLO network with deformable convolution and attention mechanisms

**DOI:** 10.1038/s41598-026-47703-3

**Published:** 2026-04-16

**Authors:** Huilin Li, Chengyang Zhang, Shaowei Ye

**Affiliations:** 1 School of Finance & Economics, Guangdong University of Science & Technology, Dongguan City, 523083 Guangdong Province China; 2 Amazon, 410 Terry Avenue North, Seattle, WA 98109 USA; 3 College of New Energy, Inner Mongolia University of Technology, Hohhot, 010051 P. R. China

**Keywords:** YOLOv11n, Pothole detection, C3k2DCY module, CDFA module, ELA-HSFPN module, Engineering, Mathematics and computing

## Abstract

Road potholes present a significant challenge to urban traffic safety and infrastructure maintenance. Traditional manual inspection methods fail to meet the demands for real-time performance and high accuracy. In this study, we propose ECC-YOLO, a lightweight object detection model based on YOLOv11n, specifically designed for pothole detection in complex road environments. First, we introduce the C3k2DCY module, which extracts multi-scale features and leverages deformable convolution to enhance the model’s ability to capture irregular geometric structures of potholes. Next, a Contrast-Driven Feature Aggregation (CDFA) module is designed to improve feature discriminability at boundary regions by reinforcing the contrast between potholes and surrounding backgrounds, thereby significantly boosting detection precision. Furthermore, an Edge-aware Lightweight Attention-based Spatial Feature Pyramid Network (ELA-HSFPN) is integrated to enable effective fusion and semantic enhancement of multi-level features, jointly combining low-level edge details and high-level semantic cues for improved localization and classification of pothole targets. Experiments conducted on a custom pothole dataset demonstrate that ECC-YOLO achieves an accuracy of 84.5%, a recall of 67.9%, and a mAP@0.5 of 74.2%, while maintaining real-time performance. Compared to the baseline YOLOv11n model, ECC-YOLO improves accuracy by 1.7% points, recall by 2.5% points, and mAP@0.5 by 1.4% points. Ablation studies further confirm the individual contribution of each module to overall performance. Overall, ECC-YOLO demonstrates excellent capability in enhancing detection accuracy, reducing false positives, and adapting to complex environments, indicating strong potential for real-world deployment.

## Introduction

Pavement surface anomalies, especially potholes, are critical factors affecting traffic safety, vehicle performance, and the sustainability of urban infrastructure^1,2^. These bowl-shaped depressions in asphalt can lead to tire blowouts and suspension system damage and pose serious safety hazards, particularly during high-speed driving, at night, or under low-light conditions. At present, many regions still rely on traditional manual inspections, which suffer from significant limitations including low efficiency, high labor costs, and susceptibility to subjective judgment. With the rapid advancement of Intelligent Transportation Systems (ITS) and autonomous driving technologies, there is an urgent need for an automated pothole detection solution that is not only accurate and real-time but also suitable for embedded deployment in complex and dynamic road environments.

Numerous researchers have proposed a variety of algorithmic frameworks targeting pothole detection, yet challenges remain in terms of adaptability, robustness, and deployment efficiency. For instance, Faisal et al.^3^ proposed a LiDAR-based approach for cost-effective quantification, but its reliance on a single data source limits generalizability across diverse road types and environmental conditions. Similarly, vision-based methods face distinct hurdles. Ouma et al.^4^ utilized clustering on low-cost 2D images, and Yousaf et al.^5^ applied traditional image processing techniques; however, both methods are highly sensitive to lighting changes and often rely on limited datasets. In the realm of deep learning, Li et al.^6^ developed a Mask R-CNN model that struggles with false positives in complex scenarios involving water or debris. Meanwhile, Fox et al.^7^ introduced a crowdsourced system, and Shi et al.^8^ designed an industrial defect algorithm, yet both approaches are often constrained by small dataset sizes. Furthermore, Salaudeen et al.^9^ attempted to improve detection through image-enhanced GANs, which introduces high computational complexity unsuitable for embedded platforms. Lastly, W. Hou et al.^10^ presented the lightweight RC-YOLOv5s model, but it still lacks precision when detecting fine or vague defects.

Despite these advancements, existing methods often suffer from unstable detection accuracy, high false positive rates, and missed detections of small targets under complex textured backgrounds. Many current models focus heavily on semantic information extraction while neglecting the fine-grained representation of edge details, resulting in poor adaptation to scenarios characterized by vague pothole contours and low boundary contrast. Methodologically, road potholes lack rigid geometric templates and frequently exhibit extremely low visual contrast against complex, weathered asphalt backgrounds. Simply integrating generic attention mechanisms or standard deformable convolutions into lightweight networks is fundamentally insufficient. Standard attention modules do not explicitly decouple the target from the background, inadvertently enhancing asphalt noise. Simultaneously, conventional deformable convolutions rely heavily on strong local feature gradients; in low-contrast scenarios, weak gradients cause the spatial sampling points to drift into the noisy background, rendering the geometric adaptation ineffective.

Therefore, the precise technical gap lies in the inability of existing methods to synergistically resolve low contrast and non-rigid deformation simultaneously. Advancing pothole detection demands a problem-driven, coupled architectural design rather than generic module substitution. To bridge this gap, this paper proposes ECC-YOLO, a model built upon an enhanced YOLOv11 architecture uniquely suited for this task. Rather than treating shape and contrast as isolated problems, ECC-YOLO establishes a sequential synergy across geometric modeling, contrast-driven feature enhancement, and multi-scale semantic fusion. The main methodological contributions of this study are as follows:

- **Explicit Foreground-Background Contrast Enhancement (via CDFA Module)**: To tackle the methodological issue of vague contours and low boundary contrast, we propose a contrast-aware feature aggregation strategy. This module explicitly decouples foreground and background structures to actively suppress redundant asphalt noise. This active noise suppression not only reduces false positives for edge-blurred targets but also generates the distinct gradient responses required for downstream spatial adaptation.
- **Spatially Adaptive Modeling for Non-Rigid Targets (via C3k2DCY Module)**: Unlike conventional convolutional structures with fixed receptive fields, we introduce a spatially adaptive methodology. Utilizing the purified gradients generated by the CDFA, the C3k2DCY module can reliably anchor its deformable sampling points onto the true irregular boundaries of potholes, flexibly perceiving spatial shifts and preventing background drift.
- **Direction-Aware Edge Semantic Fusion (via ELA-HSFPN)**: To solve the problem of traditional upsampling discarding fine-grained edge details, we design a collaborative multi-scale fusion mechanism. This approach shifts the paradigm to edge-aware semantic integration, balancing low-level textures with high-level spatial relationships to improve localization consistency for scale-inconsistent targets without sacrificing lightweight deployment efficiency.

## Related Work

### DCNv3 module

DCNv3^11^, the third-generation deformable convolutional network, represents the latest advancement in the deformable convolution series. Through a series of innovative improvements, DCNv3 significantly enhances the capability of convolutional neural networks to adapt to complex geometric transformations. Firstly, inspired by the concept of separable convolution, DCNv3 decomposes standard convolutions into depthwise convolutions and pointwise convolutions, enabling weight sharing among convolutional neurons and reducing computational complexity. In addition, it introduces a multi-group mechanism that splits the convolution process into multiple groups, each with independent offsets and feature amplitudes. This grouping strategy increases the model’s flexibility and expressive power. Moreover, DCNv3 improves the normalization scheme by replacing traditional element-wise Sigmoid normalization with Softmax normalization across sampling points, allowing the model to better capture inter-point relationships and enhance sensitivity to the geometric structure of input data. By incorporating additional offsets and modulation coefficients, DCNv3 enables convolution kernels to deform adaptively in spatial dimensions. For each target point, the model traverses all groups and the sampling points within each group to compute offset-modified features, which are then aggregated through a weighted sum using modulation scalars and projection weights. This adaptive spatial aggregation mechanism compensates for the limitations of standard convolutions in modeling long-range dependencies and performing spatially adaptive feature extraction.

DCNv3 builds upon the inductive bias of conventional convolutions while offering enhanced computational efficiency. It requires less training data and time, and outperforms earlier approaches such as large-kernel re-parameterization in both computational and memory efficiency. Owing to these advantages, DCNv3 has demonstrated exceptional performance in tasks that involve complex geometric variations, such as image segmentation, object detection, and video analysis, providing a powerful tool for various applications in the field of computer vision.

### SE attention mechanism

The SE^12^(Squeeze-and-Excitation) module is a lightweight attention mechanism designed to enhance the representational capacity of feature channels in convolutional neural networks (CNNs). Its workflow primarily consists of three steps: Squeeze, Excitation, and Scale.

In the squeeze phase, the input feature map x (with dimensions $C \times W \times H$, where c is the number of channels, and *W* and *H* are the width and height respectively) is compressed into a feature vector $C \times 1 \times 1$ through global average pooling.

In the subsequent excitation phase, two fully connected layers along with ReLU and Sigmoid activation functions are applied. First, the number of channels *C* is reduced to $C/R$ (where *R* is the reduction ratio) using the first fully connected layer to reduce computational cost. Then, a ReLU activation introduces non-linearity. The second fully connected layer restores the channel dimension to *C*, followed by a Sigmoid activation function to normalize the output between 0 and 1, resulting in an importance weight vector $C \times 1 \times 1$ for each channel.

Finally, during the scale phase, the weights obtained in the excitation step are applied to the original input feature map *X* via channel-wise multiplication. In this way, important channel features are enhanced while less relevant ones are suppressed, thereby optimizing feature representation. This allows the network to allocate its limited resources more effectively and improve overall model performance.

Due to its lightweight design, general applicability, and learnable nature, the SE module has been widely adopted in tasks such as image classification^13^, object detection^14^, and semantic segmentation^15^.

### Advances in Lightweight Object Detection

Recent years have witnessed rapid advancements in lightweight object detection, primarily driven by the evolution of the YOLO series and the introduction of efficient Transformer-based architectures. Recent YOLO variants, such as YOLOv8, YOLOv10, and the baseline YOLOv11, have significantly optimized the balance between detection accuracy and inference speed through advanced label assignment strategies and streamlined architectures. However, fundamentally, these models still heavily rely on rigid convolutional grids. This architectural trait makes them inherently constrained when attempting to capture the highly irregular, non-rigid morphological features of road potholes, particularly in scenarios where the target shares a highly similar texture with the background. Simultaneously, efficient Transformer-based detectors, such as RT-DETR, achieve remarkable global context modeling through self-attention mechanisms. Despite this, Transformers typically exhibit weaker local inductive biases, which often results in suboptimal boundary alignment for small, edge-blurred targets in pothole detection. Furthermore, the inherent computational complexity of self-attention mechanisms poses a higher computational burden, challenging seamless deployment on resource-constrained road maintenance edge devices.

To address multi-scale semantic integration, modern lightweight feature fusion architectures (e.g., PANet, BiFPN) have been widely adopted. Yet, their conventional isotropic upsampling operations tend to physically dilute fine-grained edge semantics during feature expansion. This dilution is particularly detrimental for pothole detection, where boundary contrast is already severely degraded by complex lighting and weathered asphalt textures. Within the specific domain of infrastructure inspection, researchers have extensively explored these improved architectures and feature fusion strategies. For instance, ultra-lightweight models incorporating bidirectional multi-scale spatial pyramids (e.g., YOLO-ROC^16^ and dynamic scale-aware fusion networks (e.g., RT-DSAFDet^17^ have been proposed to improve real-time road damage detection on edge devices. Additionally, multi-scale dual-attention networks (such as EMDANet^18^ and lightweight segmentation frameworks (like GTRS-Net^19^ and ERCU-Net^20^ have been developed to enhance the extraction of fine-grained crack and pothole margins under complex UAV and ADAS perspectives.

Critical Synthesis: Comprehensive surveys of these methodologies highlight a continuous trade-off between computational efficiency and detection robustness^21^. A deeper comparative analysis of these state-of-the-art approaches reveals a common unresolved limitation: they generally treat spatial adaptation, contrast enhancement, and multi-scale feature fusion as isolated optimization tasks. While they successfully utilize generic attention and feature compression to reduce parameters, they fail to synergistically address the coupled challenges of low-contrast backgrounds and severe irregular geometric deformations. Specifically, they lack a dedicated mechanism to explicitly decouple the unique low-contrast background noise inherent to weathered asphalt before applying spatial convolutions. The proposed ECC-YOLO framework is necessitated specifically to bridge this gap by establishing a holistic, problem-driven sequential synergy. By tightly coupling explicit background suppression (via the CDFA module to create clean gradient fields) with spatially adaptive geometric modeling (via the C3k2DCY module to anchor sampling points onto irregular boundaries) and concluding with anisotropic, direction-aware edge fusion (via the ELA-HSFPN module), ECC-YOLO uniquely provides an architecture that current generic lightweight SOTA models lack.

## Method design

**Fig. 1:**
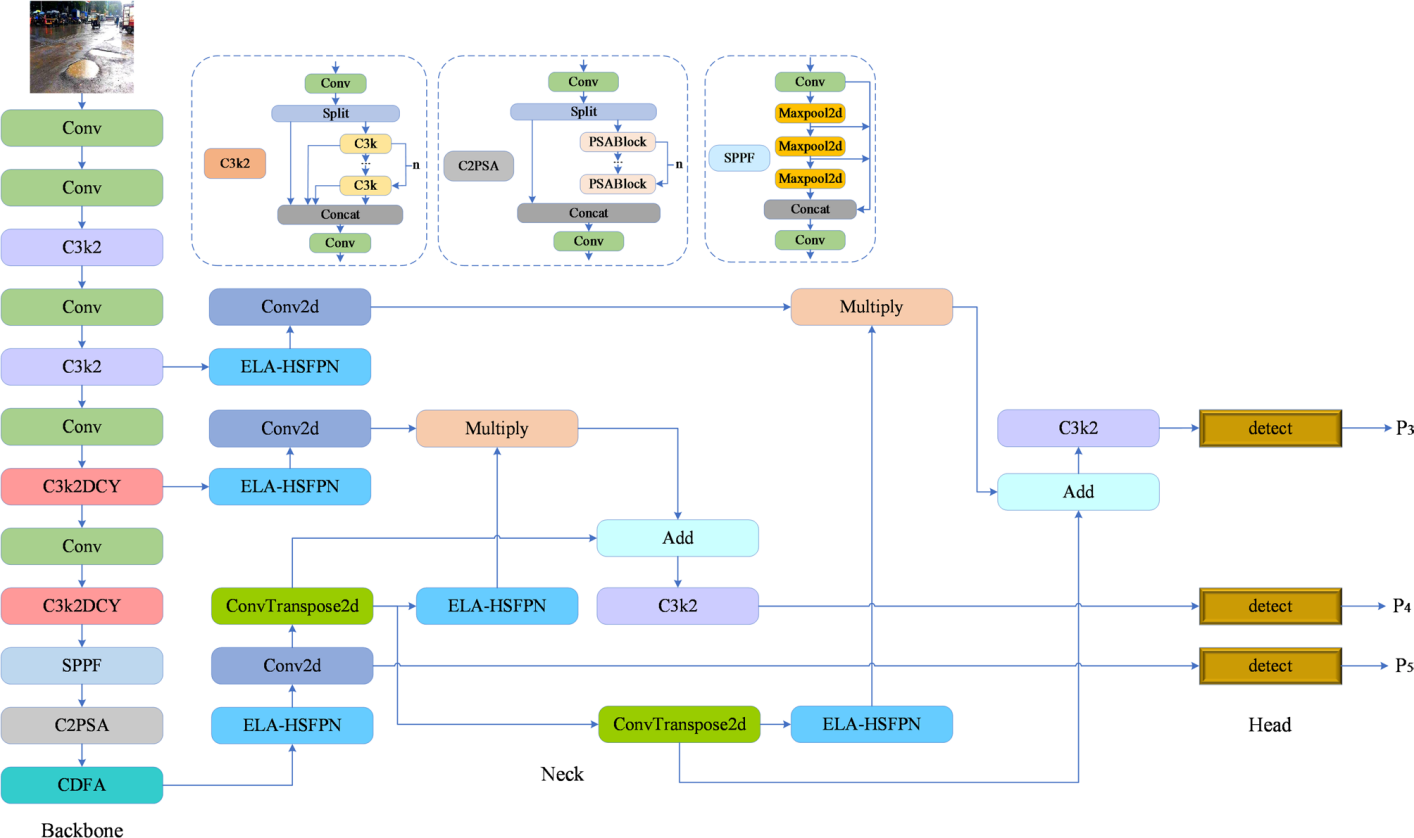
shows the improved network structure of YOLOv11n.

This chapter provides a detailed introduction to the proposed ECC-YOLO model architecture and its key components. Based on the lightweight YOLOv11n framework^22^, ECC-YOLO integrates three core innovative modules: the C3k2DCY module, which enhances the model’s adaptability to geometric deformations; the Contrast-Driven Feature Aggregation (CDFA) module, which improves edge and foreground recognition; and the Edge-aware Lightweight Spatial Feature Pyramid Network (ELA-HSFPN), which enables efficient, direction-aware multi-scale feature fusion. The overall architecture of the model is shown in Fig. 1.

### C3k2DCY module

To enhance the lightweight detection model’s ability to represent irregular targets such as potholes, this paper proposes the C3k2DCY module. Built upon the YOLOv11n backbone, this structure integrates the spatially adaptive DCNv2-MPCA units^23,24^, and constructs a multi-level feature enhancement path from Bottleneck to C3k, and ultimately to C3k2.

**Fig. 2:**
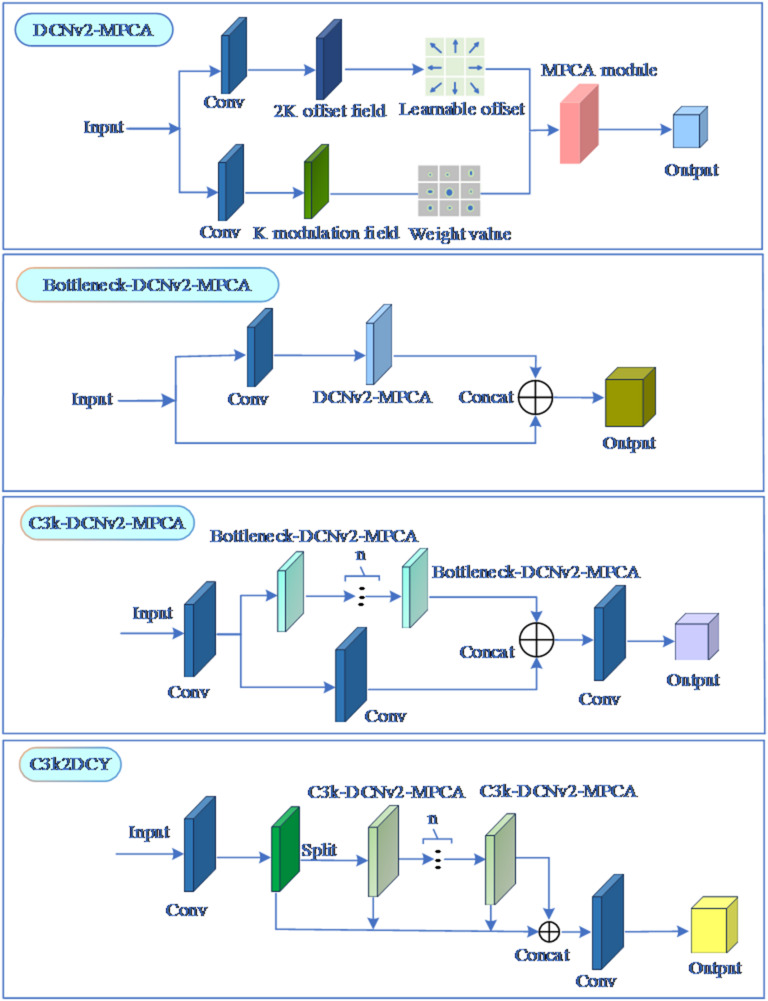
C3k2DCY module.

As illustrated in Fig. 2, the DCNv2-MPCA unit employs a dual-branch convolution to learn spatial offsets and modulation masks separately, and leverages the MPCA module to perform dynamic modulation of sampling points, thereby enhancing the model’s responsiveness to object boundaries. On this basis, the Bottleneck-DCNv2-MPCA structure embeds deformable convolution into residual units and combines residual connections to improve the stability of deep feature extraction. Furthermore, the C3k structure extracts high-order nonlinear features through the stacked Bottleneck units, while the final C3k2 structure adopts a strategy of feature partitioning, dual-path parallel processing, and cross-scale fusion, effectively combining shallow and deep receptive fields to boost the model’s perception of complex pothole structures. Unlike existing YOLO series models that rely on fixed receptive fields and single-scale convolutional processing, the C3k2DCY module achieves spatially adaptive modeling, multi-path fusion, and boundary alignment enhancement through synergistic optimization. With only a minimal increase in parameters, it significantly improves detection accuracy and boundary fitting performance for non-rigid targets, demonstrating clear structural innovation and practical deployment value.

Behaviorally, the C3k2DCY module fundamentally alters how the network samples image context. When a standard rigid convolutional layer encounters an irregularly shaped pothole, its fixed square receptive field inevitably samples a significant amount of irrelevant background asphalt, diluting the target features. In contrast, the C3k2DCY module dynamically adjusts its spatial sampling grid. Driven by the learned offsets, the sampling points actively warp and cluster around the pothole’s unique contour. This behavior ensures that the feature maps passed to deeper layers are highly target-specific and free from background pollution, enabling the network to accurately perceive severe morphological deformations.

### CDFA module

To enhance the model’s ability to distinguish pothole edges under complex textures and low-contrast backgrounds, this paper proposes the Contrast-Driven Feature Aggregation (CDFA) module. This module integrates multi-scale context enhancement, wavelet-based feature decoupling, and a foreground-background dual-path attention mechanism, establishing a contrast-aware feature aggregation strategy. The core idea is to explicitly decouple foreground and background structures when fusing feature maps of different semantic levels, constructing local contrast-aware windows to guide feature enhancement in high-response regions while suppressing redundant background activations.

**Fig. 3:**
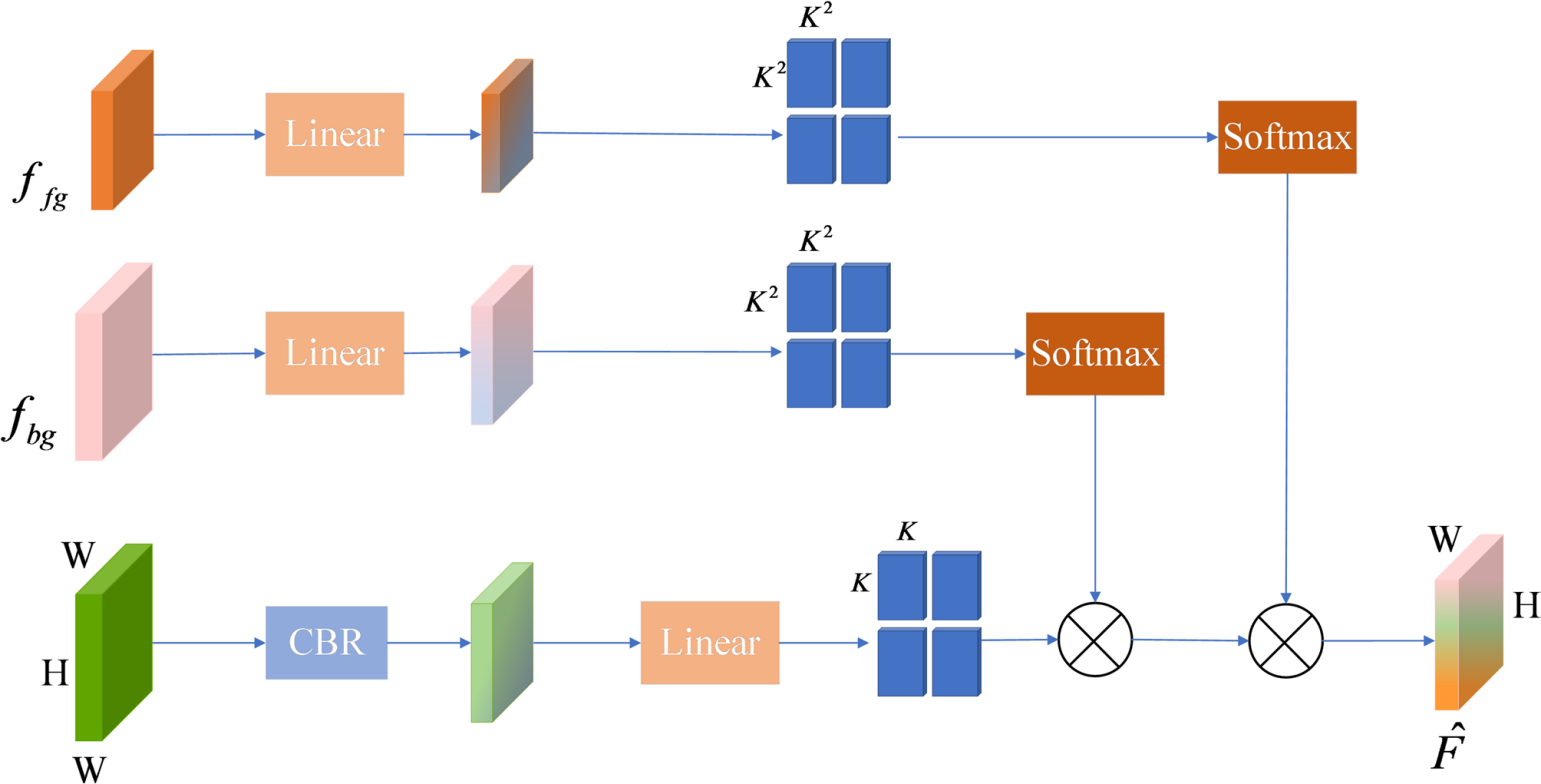
CDFA attention mechanism.

Figure 3 illustrates the structure of the CDFA^25^(Contrast-Driven Feature Aggregation) module. Its core idea is to leverage the contextual differences between foreground and background to achieve dynamic feature aggregation within local spatial regions. Specifically, CDFA first decouples the deep features into a foreground feature map ${f_{fg}}$ and a background feature map ${f_{bg}}$. For any spatial location $(i,j)$, CDFA calculates attention weights within a local window $K \times K$ centered on that position, thereby integrating contextual information from neighboring regions. Initially, the input feature map $F \in {{\mathbb{R}}^{H \times W \times C}}$ is passed through several CBR (Convolutional Block with ReLU) modules, which include convolution, BatchNorm, and ReLU operations, to perform preliminary feature fusion and generate a value vector $V \in {{\mathbb{R}}^{H \times W \times C}}$ of dimension ${\mathrm{C}}$. This vector is then processed through a linear layer with weights ${W_v} \in {{\mathbb{R}}^{C \times C}}$. The resulting value vector *V* is expanded within each local window to prepare for aggregating neighborhood information at each position. Let ${V_{{{{\boldsymbol{\Delta}}}_{i,j}}}} \in {{\mathbb{R}}^{C \times {K^2}}}$ denote all the values within the local window centered at $(i,j)$, defined as:

1 $${V_{{{{\boldsymbol{\Delta}}}_{i,j}}}}=\left\{ {{V_{i+p - \left\lfloor {\frac{K}{2}} \right\rfloor ,j+q - \left\lfloor {\frac{K}{2}} \right\rfloor }}} \right\},0 \leqslant p,q<K.$$

The foreground feature map ${f_{fg}}$ and the background feature map ${f_{bg}}$ are processed separately through two distinct linear layers to generate the corresponding attention weights ${A_{fg}} \in {{\mathbb{R}}^{H \times W \times {K^2}}}$ and ${A_{bg}} \in {{\mathbb{R}}^{H \times W \times {K^2}}}$, respectively. The calculation of the attention weights can be expressed as:

2 $${A_{fg}}={W_{fg}} \cdot {f_{fg}}\quad$$

3 $${A_{bg}}={W_{bg}} \cdot {f_{bg}}$$

Among them, ${W_{fg}} \in {{\mathbb{R}}^{C \times {K^4}}}$ and ${W_{bg}} \in {{\mathbb{R}}^{C \times {K^4}}}$ are the weight matrices of the linear transformations applied to the foreground and background feature maps, respectively. Subsequently, the foreground and background attention weights at position $(i,j)$ are reshaped into ${\hat {A}_{f{g_{i,j}}}} \in {{\mathbb{R}}^{{K^2} \times {K^2}}}$ and ${\hat {A}_{b{g_{i,j}}}} \in {{\mathbb{R}}^{{K^2} \times {K^2}}}$, and both are activated using the Softmax function. Then, the unfolded value vector ${\hat {V}_{{{{\boldsymbol{\Delta}}}_{i,j}}}}$ is weighted in two steps:

4 $${\hat {V}_{{{{\boldsymbol{\Delta}}}_{i,j}}}}={\mathrm{Softmax}}({\hat {A}_{f{g_{i,j}}}}) \otimes ({\mathrm{Softmax}}({\hat {A}_{b{g_{i,j}}}}) \otimes {V_{{{{\boldsymbol{\Delta}}}_{i,j}}}}).$$

Here, $\otimes$ denotes matrix multiplication. Finally, the weighted value representations are densely aggregated to obtain the final output feature map. Specifically, the aggregated feature at position $(i,j)$ is:

5 $${\hat F_{i,j}} = \mathop \sum \limits_{0 \leqslant m,n < K} \hat V_{{{{\Delta }}_{i + m - \left\lfloor {\frac{K}{2}} \right\rfloor ,j + n - \left\lfloor {\frac{K}{2}} \right\rfloor }}}^{i,j}$$

From a detection behavior perspective, the CDFA module functions as an active noise-suppression and edge-enhancement mechanism. In complex road environments, potholes often share highly similar color and texture with the surrounding weathered asphalt, confusing standard feature extractors. The CDFA alters the network’s behavior by creating a competitive dynamic between the foreground and background. When calculating attention weights, it actively dampens the feature activations of flat, uniform asphalt regions (background) while simultaneously amplifying the responses at gradient shifts and texture anomalies (foreground edges). This behavioral shift prevents the network from over-responding to road patches or shadows, directly addressing the high false-positive rate associated with low-contrast targets.

### ELA-HSFPN module

To achieve efficient fusion of multi-scale features and enhance edge refinement, this paper proposes the Edge-aware Lightweight Spatial Feature Pyramid Network (ELA-HSFPN) to replace the traditional upsampling and concatenation mechanism in the YOLO architecture. This module maintains high computational efficiency while improving adaptability to pothole edges, texture directionality, and scale-inconsistent targets.

**Fig. 4:**
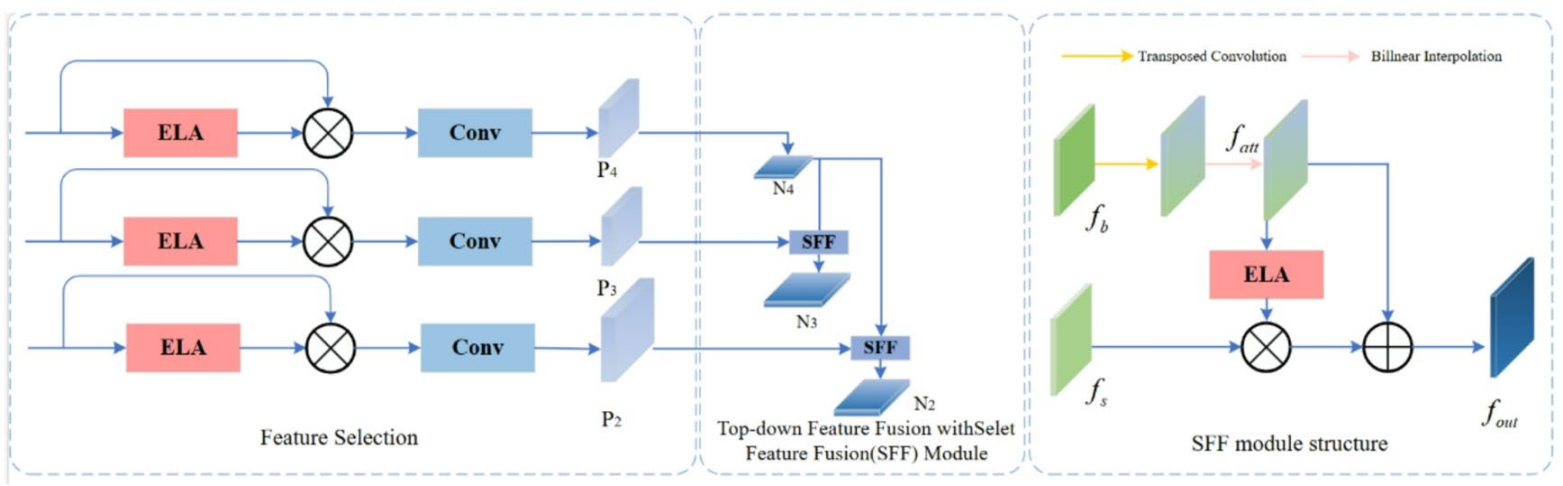
ELA-HSFPN module.

Figure 4 illustrates the structure of the ELA-HSFPN^26^ module. The process of the ELA module is as follows: First, the input feature map undergoes one-dimensional global average pooling in both the horizontal and vertical directions to extract global contextual information along each axis. Then, the pooled results are separately encoded through one-dimensional convolutions and group normalization to generate positional attention maps for the horizontal and vertical directions, respectively. Finally, these two positional attention maps are element-wise multiplied with the original feature map, thereby enhancing spatial position awareness of the features.

In the feature fusion stage, the SFF (Selective Feature Fusion) module is employed. First, the large-scale feature ${f_b} \in {{\mathbb{R}}^{C \times H \times W}}$ and the small-scale feature ${f_s} \in {{\mathbb{R}}^{C \times H \times W}}$ are input into the module. Next, the large-scale feature ${f_b}$ is upsampled using a transposed convolution ${T_{{\mathrm{conv}}}}$ with a kernel size of $3 \times 3$ and a stride of 2, resulting in the expanded feature ${f_b} \in {{\mathbb{R}}^{C \times H \times W}}$. The expanded feature is then integrated with the small-scale feature. Subsequently, the ELA module divides the spatial dimensions into horizontal and vertical directions to capture directional feature information. Finally, the captured features are fused to produce the output feature ${f_{{\mathrm{out}}}} \in {{\mathbb{R}}^{C \times {H_1} \times {W_1}}}$. Equations (7) and (8) describe the specific process of feature selection and fusion:

7 $${f_{{\mathrm{att}}}}=BL({T_{{\mathrm{conv}}}}({f_b}))$$

8 $${f_{{\mathrm{out}}}}={f_s} \times ELA({f_{{\mathrm{att}}}})+{f_{{\mathrm{att}}}}$$

The behavioral impact of the ELA-HSFPN module becomes most evident during the localization of scale-inconsistent potholes. Traditional upsampling behaves isotropically, uniformly expanding feature maps, which physically blurs and dilutes the sharp boundary semantics required for precise bounding box regression. By integrating horizontal and vertical positional attention, the ELA-HSFPN forces the fusion process to behave anisotropically. It explicitly preserves the directional gradient information of the pothole edges during multi-scale integration. Consequently, the network behaves with much higher boundary sensitivity, producing significantly tighter and more accurate bounding boxes, especially for small-scale potholes whose edge features would otherwise be washed out.

### Architectural synergy and conceptual contribution

While individual mechanisms like attention and deformable convolutions have been explored in general object detection, their naive integration often yields suboptimal results for pothole detection. This limitation stems from the specific physical characteristics of road anomalies: extremely low foreground-background contrast coupled with severe non-rigid geometric deformation. Therefore, the conceptual contribution of the ECC-YOLO framework extends beyond empirical module integration. It establishes a tightly coupled, non-redundant feature representation pipeline specifically tailored to resolve these dual challenges.

The specific combination of CDFA, C3k2DCY, and ELA-HSFPN is optimal because these modules operate synergistically across distinct feature processing domains (magnitude, spatial geometry, and scale topology), ensuring zero functional redundancy. They form a sequential, interdependent pipeline:

- **The Pre-conditioner (CDFA)**: Standard deformable convolutions often fail in pothole detection because weak feature gradients in weathered asphalt cause spatial sampling points to drift chaotically into the background. To resolve this, the CDFA module explicitly decouples the foreground and background, actively enhancing boundary contrast and forcing the generation of sharp gradient responses.
- **The Geometric Adapter (C3k2DCY)**: Relying strictly on these purified, high-contrast gradients, the C3k2DCY module effectively anchors its deformable sampling points onto the true irregular boundaries of the pothole, completely avoiding the background drift that plagues conventional networks.
- **The Semantic Preserver (ELA-HSFPN)**: Finally, this module guarantees that the precisely extracted, shape-adaptive local features are fused anisotropically across multiple network scales. This prevents the physical dilution of fine-grained edge semantics that inevitably occurs during traditional isotropic upsampling.

Consequently, the proposed architecture provides a unified, problem-driven feature representation principle: active contrast enhancement (CDFA) enables accurate spatial deformation (C3k2DCY), and the resulting features are seamlessly preserved via direction-aware scale fusion (ELA-HSFPN). This joint contribution forms a complete, logical closed loop tailored specifically for robust road anomaly detection.

## Experiment

### Dataset

**Fig. 5:**
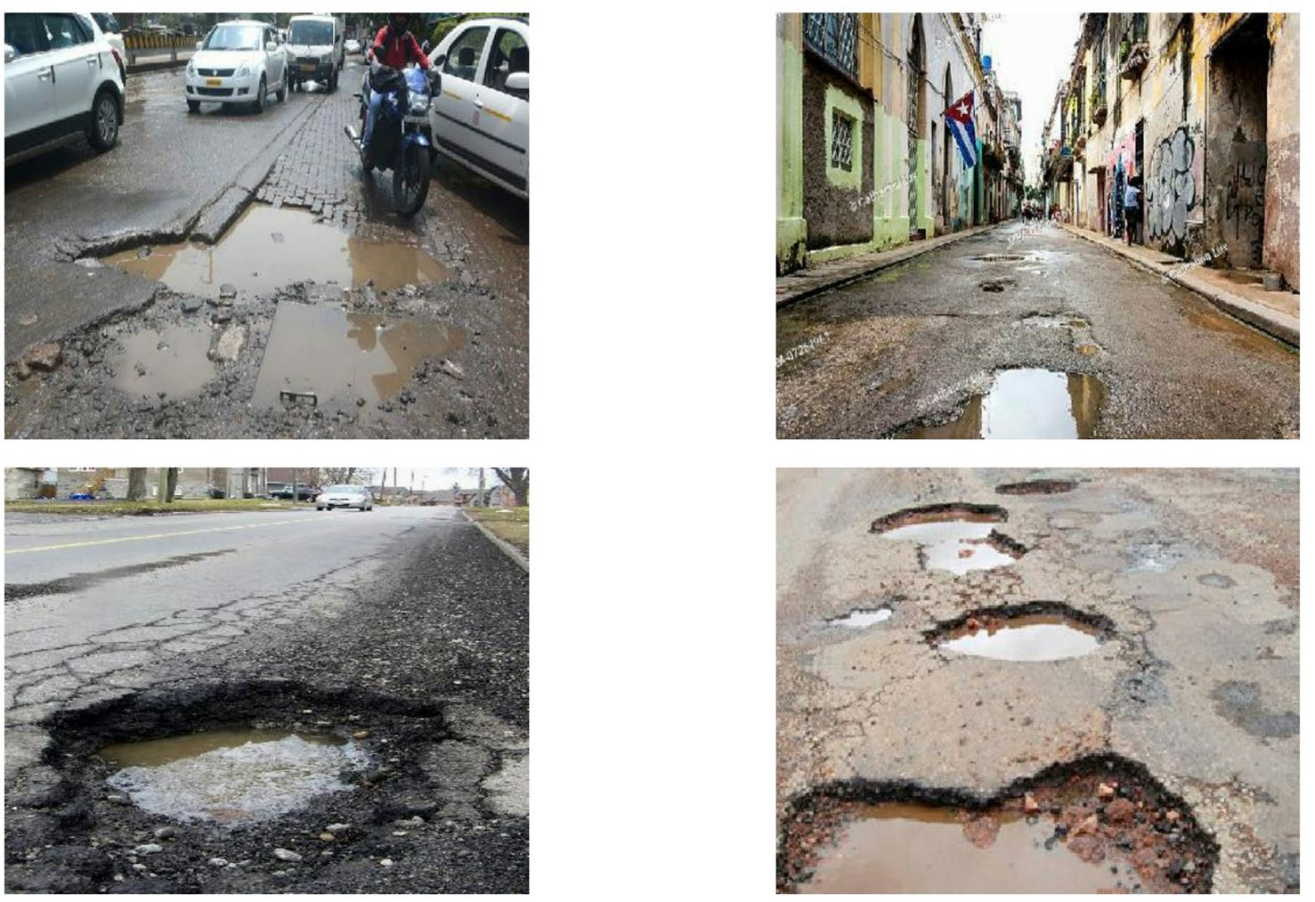
Image of pit defect.

This study utilized a publicly available pothole dataset provided by the Roboflow platform as the experimental data source^27^. The dataset focuses on the detection of pothole defects in real-world road environments, covering various types of road surfaces and lighting conditions, offering considerable complexity and realistic representation. As shown in Fig. 5, the images in the dataset exhibit typical variations in pothole shapes and are often accompanied by challenging factors such as background interference, object occlusion, and blurred edges, making detection more difficult. The original dataset contains a total of 665 images, all captured from real road scenarios. The dataset was split in a 7:2:1 ratio into a training set, validation set, and test set, consisting of 465, 66, and 134 images respectively.

To enhance the model’s generalization ability and robustness in complex scenarios, various data augmentation techniques were applied to the training set. These included image flipping, brightness perturbation, scaling and cropping, rotation and translation, salt-and-pepper noise addition, occlusion simulation, and grayscale transformation. All augmentation operations were performed using probability-controlled random mechanisms to increase the diversity of training samples and improve distribution generalization. During augmentation, each original training image was used to generate four augmented samples, resulting in 1,860 augmented images. Combined with the original training images, the final training set consisted of 2,325 images. Throughout the augmentation process, all label information was strictly aligned with the original images, ensuring that the augmented samples remained consistent with the real-world scenarios both spatially and semantically. This effectively improved the model’s detection performance on pothole targets with weak boundaries, low contrast, and complex backgrounds.

### Experimental platform

To ensure efficient execution of the model training process and stability of the results, the experiments in this study were conducted on a high-performance software and hardware platform. The operating system, programming environment, deep learning framework, and hardware devices were all properly configured to meet the computational demands of large-scale image processing and deep neural network training. The detailed experimental platform configuration is shown in Table 1.

**Table 1:** Experimental Platform Configuration.

Item	Configuration
Operating System	Ubuntu 20.04
Programming Language	Python 3.8
Deep Learning Framework	PyTorch 2.0.0 + CUDA 11.8
GPU	NVIDIA RTX 4090 (24 GB VRAM)
CPU	AMD EPYC 7T83 (22 cores)

### Hyperparameter setting

In terms of training configuration, the input image size was uniformly set to 640 × 640, with a batch size of 64 and a total of 200 training epochs. The initial learning rate was set to 0.01, the momentum factor to 0.937, and the weight decay coefficient to 0.0005, aiming to achieve effective gradient optimization and regularization control.

During data loading, 4 worker threads were enabled to improve image reading and preprocessing efficiency. Under the specified hardware and hyperparameter configurations, the total training time for the ECC-YOLO model over 200 epochs was approximately 0.5 h. These training hyperparameters were optimized through multiple rounds of preliminary experiments to ensure stable convergence while balancing model performance and training efficiency.

### Evaluation Indicators

To comprehensively evaluate the detection performance of the model, this study adopts F1 score, Precision (P), Recall (R), Average Precision (AP), and mean Average Precision (mAP) as the main evaluation metrics^28^. In addition, the number of parameters (Parameters) is also considered to assess the model’s complexity and deployment efficiency. The formulas for the above metrics are defined as follows:

9 $${\mathrm{Precision}}=\frac{{T{\mathrm{p}}}}{{T{\mathrm{p}}+F{\mathrm{p}}}},$$

10 $${\mathrm{Recall}}=\frac{{T{\mathrm{p}}}}{{T{\mathrm{p}}+F{\mathrm{N}}}},$$

11 $${\mathrm{AP}}=\int_{0}^{1} {P(R){\mathrm{d}}R} ,$$

12 $${\mathrm{mAP}}=\frac{1}{n}\sum\limits_{{{\mathrm{i}}=0}}^{n} {AP(i)} ,$$

13 $${\mathrm{F}}1=\frac{{2 \times {\mathrm{Precision}} \times {\mathrm{Recall}}}}{{{\mathrm{Precision}}+{\mathrm{Recall}}}},$$

Where $T{\mathrm{p}}$ denotes the number of correctly detected defect targets; $F{\mathrm{p}}$ represents the number of falsely detected defect targets; $F{\mathrm{N}}$ indicates the number of missed defect targets; n refers to the number of object classes; and $AP(i)$ represents the average precision for the i-th object class.

## Experimental analysis

### Algorithm comparison results

To evaluate the performance and robustness of the proposed ECC-YOLO in pothole detection, we systematically compared it with several mainstream object detection methods. The baseline models include widely adopted lightweight YOLO architectures (YOLOv5n^29^, YOLOv5s, YOLOv7-tiny^30^, YOLOv8n^31^, YOLOv10n^32^, YOLOv10s, and YOLOv11n) and RT-DETR-r18^33^, a representative Transformer-based architecture. All models were tested under identical dataset and training configurations. Their performances were comprehensively evaluated based on detection accuracy, boundary fitting ability, and parameter size to validate the advantages of our proposed method.

**Table 2:** Comparison of key metrics across different algorithms.

Algorithm	Precision/%	Recall/%	mAP@0.5/%	mAP@0.5:0.95/%
YOLOv5n	77.6	62.5	69.0	35.0
YOLOv5s	75.2	61.2	66.6	35.0
YOLOv7-tiny	77.4	65.8	73.0	38.7
YOLOv8n	79.2	58.6	64.2	35.4
YOLOv10n	70.6	52.4	59.5	33.5
YOLOv10s	78.5	49.3	58.2	33.3
RT-DETR-r18	78.5	62.5	72.6	42.0
YOLOv11n	82.8	65.4	72.8	41.8
ECC-YOLO	84.5	67.9	74.2	42.6

Based on the overall results in Table 2, ECC-YOLO achieved the best performance across all four core metrics: Precision, Recall, mAP@0.5, and mAP@0.5:0.95. Compared to the baseline YOLOv11n, ECC-YOLO improved mAP@0.5:0.95 by 0.8% and Recall by 2.5%, demonstrating the effectiveness of the collaborative optimization among its modules. Relative to YOLOv7-tiny, ECC-YOLO showed a 7.1% increase in Precision and a 3.9% improvement in mAP@0.5:0.95. Although the YOLOv5 and YOLOv10 series offer advantages in lightweight design, their Precision and Recall were generally lower for this task. In particular, YOLOv10n/s exhibited weak detection capabilities under complex backgrounds, with mAP@0.5:0.95 scores below 34%. While YOLOv8n slightly outperformed YOLOv5 in certain metrics, its overall mAP performance was inferior to that of YOLOv7-tiny and YOLOv11n. Notably, while the Transformer-based RT-DETR-r18 achieved a competitive mAP@0.5:0.95 of 42.0%, its Precision and Recall were both lower than those of ECC-YOLO, indicating trade-offs between boundary modeling and practical lightweight efficiency.

To ensure the statistical reliability of these improvements and eliminate the influence of training variability, we conducted five independent training runs for both the baseline YOLOv11n and the proposed ECC-YOLO. The baseline model achieved a mean mAP@0.5 of 72.82% (± 0.18%), whereas ECC-YOLO achieved 74.21% (± 0.15%). These exceptionally narrow standard deviations confirm that the model’s convergence is highly stable, statistically validating that the performance gain is robust and fundamentally driven by the architectural enhancements rather than random initialization noise.

Furthermore, while the quantitative performance gains over the baseline YOLOv11n may appear moderate in absolute terms, they represent a highly efficient balance between architectural complexity and detection capability. Because the baseline YOLOv11n is already a rigorously optimized lightweight network, securing further accuracy improvements in highly complex, low-contrast scenarios typically dictates scaling up to much larger model parameters. Such brute-force scaling inevitably sacrifices the real-time processing feasibility required for embedded deployment in Intelligent Transportation Systems (ITS). ECC-YOLO deliberately circumvents this limitation. The introduced components are strategically designed to inflict minimal computational overhead while effectively resolving specific, critical bottlenecks, namely eliminating background false positives and reinforcing boundary sensitivity. Consequently, this targeted architectural refinement translates the moderate numerical gains into a substantial improvement in real-world robustness, perfectly aligning with the stringent latency and resource constraints of edge-device deployment.

**Table 3:** Comparison of computational efficiency metrics.

Algorithm	FPS	GFLOPs	Params (M)
YOLOv5s	147.1	15.8	7.01
YOLOv7-tiny	151.5	13.0	6.01
YOLOv10s	128.2	24.4	8.03
RT-DETR-r18	64.9	56.9	19.87
ECC-YOLO	153.8	7.9	5.41

Furthermore, providing a clear analysis of the trade-off between detection accuracy and computational cost is essential to support the claim of real-time lightweight performance. As detailed in Table 3, the proposed ECC-YOLO successfully avoids brute-force network scaling. It achieves optimal detection performance with an exceptionally low computational overhead of only 5.41 M parameters and 7.9 GFLOPs. More importantly, during testing, ECC-YOLO maintained an outstanding inference speed of 153.8 FPS. The model fundamentally retains ultra-lightweight characteristics. For instance, its 153.8 FPS significantly outperforms heavier architectures like YOLOv10s and RT-DETR-r18, and it is even faster than classic lightweight models such as YOLOv5s and YOLOv7-tiny, all while delivering superior boundary sensitivity and accuracy. This specific architectural refinement translates precision gains into substantial real-world robustness, flawlessly aligning with the stringent latency and computational constraints required for real-time edge-device deployment in Intelligent Transportation Systems.

### Results visualization

**Fig. 6:**
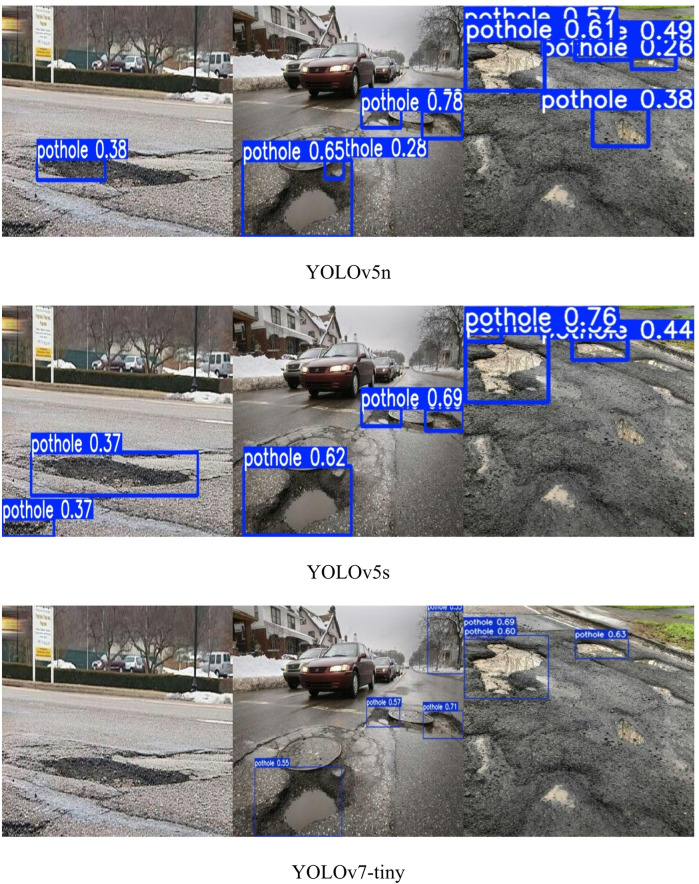
presents the prediction results of YOLOv5n, YOLOv5s, and YOLOv7-tiny in typical road scenarios.

**Fig. 7:**
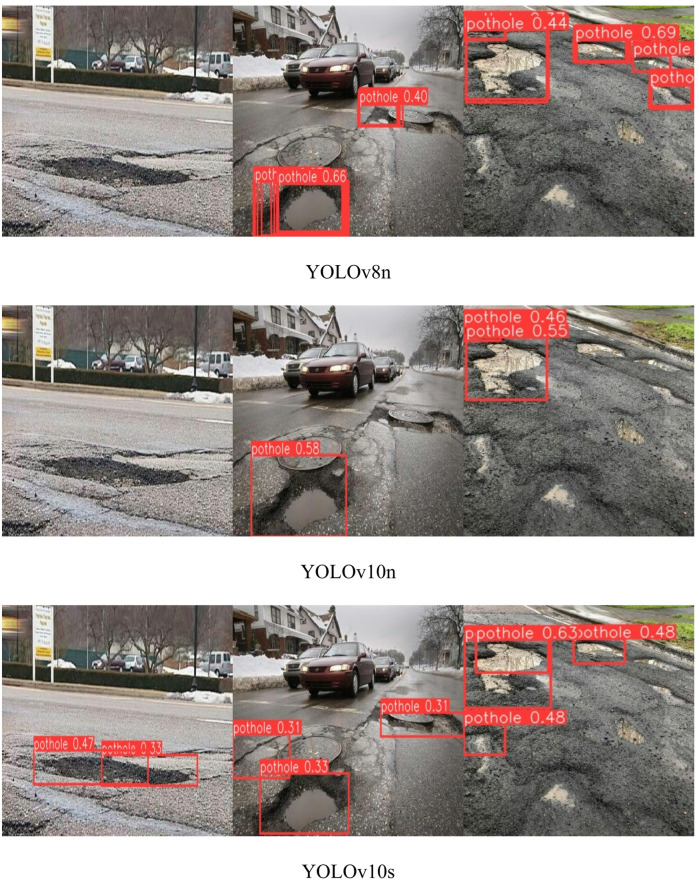
presents the prediction results of YOLOv8n, YOLOv10n, and YOLOv10s in typical road scenarios.

**Fig. 8:**
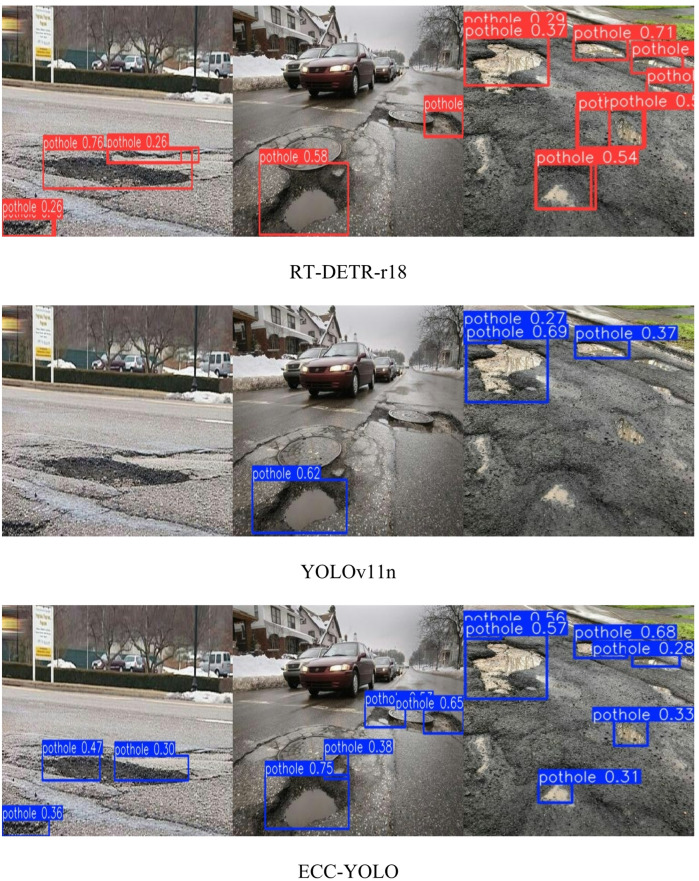
presents the prediction results of RT-DETR-r18, YOLOv11n, and ECC-YOLO in typical road scenarios.

To further validate the model’s object perception capability and detection performance in real-world scenarios, representative image samples were selected for visual analysis. By comparing the predicted bounding boxes, object confidence scores, and boundary alignment of each model on the same images, it is possible to more intuitively assess their ability to detect pothole targets at different scales, adapt to complex backgrounds, and identify potential missed detections. This visualization process not only effectively complements the quantitative results presented in the previous tables but also provides intuitive evidence to illustrate the practical advantages brought by the proposed structural improvements. Figures 6 and 7, and 8 show the detection results of all comparison models on road surface images.

The visualization results across complex road scenarios reveal the intrinsic architectural limitations of conventional lightweight models. Earlier iterations, such as the YOLOv5, YOLOv7, and YOLOv8 series (Figs. 6 and 7), exhibit varying degrees of false negatives (missed small targets) and false positives (redundant or misplaced bounding boxes). Analytically, this behavior stems from their reliance on standard convolutions and conventional feature pyramids, which struggle to decouple foreground potholes from the highly similar textures of weathered background asphalt. For instance, YOLOv8n and YOLOv10s over-respond to ambiguous textures, causing severe bounding box redundancy, while YOLOv10n suffers from feature dilution, leading to extensive missed detections of low-contrast targets.

While RT-DETR-r18 (Fig. 8) demonstrates robust global perception due to its Transformer architecture, it still produces overlapping boxes, revealing a weakness in fine-grained local boundary sensitivity. The baseline model, YOLOv11n, provides a relatively balanced output but still fails to consistently enclose small, irregular targets with tight boundaries, primarily because standard upsampling behaves isotropically and blurs edge semantics.

In contrast, the proposed ECC-YOLO demonstrates superior analytical performance that directly correlates with its methodological innovations. The visual absence of background misclassifications (false positives) validates the effectiveness of the CDFA module, which actively suppresses flat asphalt responses and amplifies gradient shifts at the pothole edges. Furthermore, the bounding boxes generated by ECC-YOLO are noticeably tighter and perfectly aligned with highly irregular pothole contours. This visually confirms the adaptive spatial sampling capability of the C3k2DCY module and the direction-aware edge preservation driven by the ELA-HSFPN. Consequently, ECC-YOLO successfully translates architectural enhancements into highly robust and precise visual detection outputs under complex real-world conditions.

**Fig. 9:**
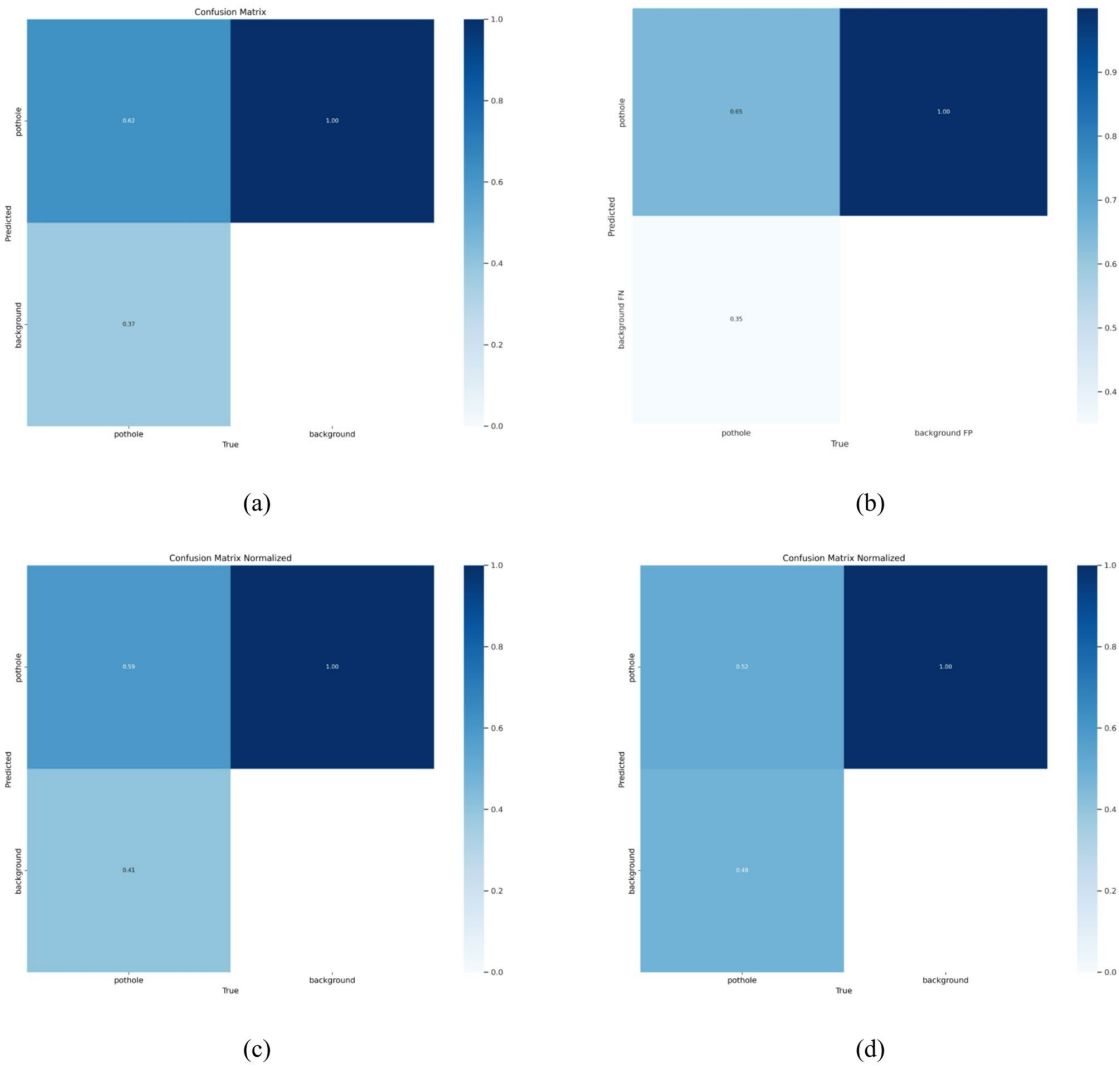

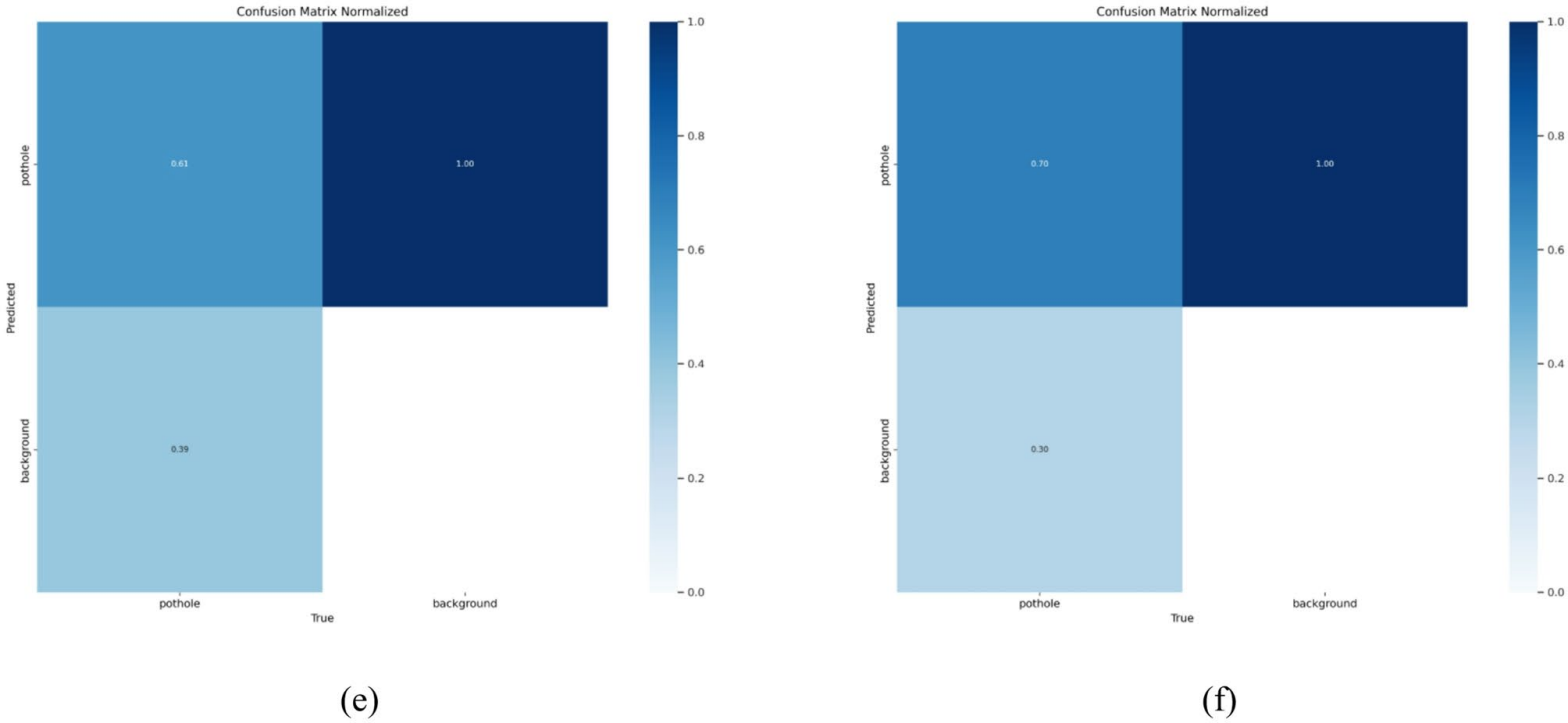
Normalized Confusion Matrices: (**a**) YOLOv5n; (**b**) YOLOv7-tiny; (**c**) YOLOv8n; (**d**) YOLOv10n; (**e**) YOLOv11n; (**f**) ECC-YOLO.

To further analyze the specific performance of each model in terms of class discrimination, this study introduces the confusion matrix as a supplementary analytical tool. It provides an intuitive representation of each model’s ability to distinguish between the “pothole” and “background” classes, as well as the degree of misclassification. As shown in Fig. 9, the six normalized confusion matrices correspond to YOLOv5n, YOLOv7-tiny, YOLOv8n, YOLOv10n, YOLOv11n, and the proposed ECC-YOLO model.

In Fig. 9(a), YOLOv5n achieves a pothole recognition accuracy of 0.62, but its high background misclassification rate of 0.37 indicates it struggles with complex textures or blurred boundaries. YOLOv7-tiny in Fig. 9(b) achieves a slightly higher recognition rate of 0.65, yet its 0.35 misclassification rate suggests the improvement in robustness is limited. YOLOv8n and YOLOv10n (Figs. 9(c) and 9(d)) exhibit further performance declines, with recognition rates dropping to 0.59 and 0.52, and misclassification rates rising to 0.41 and 0.48, respectively. These results indicate that structural simplification significantly impaired their ability to accurately distinguish fine-grained road targets, particularly in cases with ambiguous boundaries. As shown in Fig. 9(e), YOLOv11n demonstrates a slight recovery with a recognition rate of 0.61, but its 0.39 misclassification rate suggests redundant responses in complex scenes remain difficult to eliminate. In contrast, Fig. 9(f) shows that ECC-YOLO delivers the best performance, achieving a recognition rate of 0.70 and the lowest background misclassification rate of 0.30. These results strongly validate the synergistic effect of the proposed C3k2DCY, CDFA, and ELA-HSFPN modules in enhancing foreground feature discrimination and suppressing background noise. In summary, this comparison confirms ECC-YOLO’s comprehensive advantages, significantly improving both recognition accuracy and adaptability in complex real-world scenarios.

**Fig. 10:**
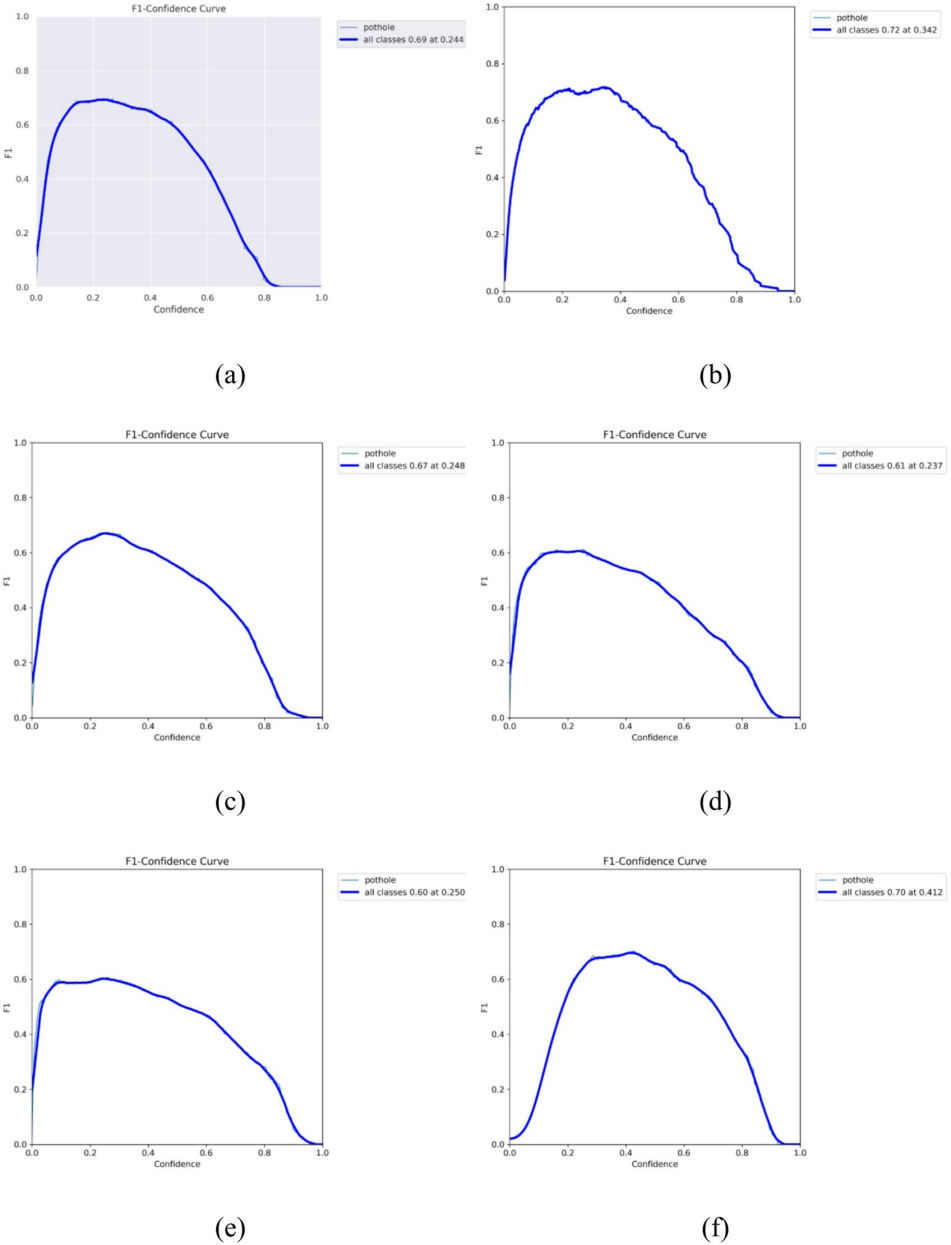

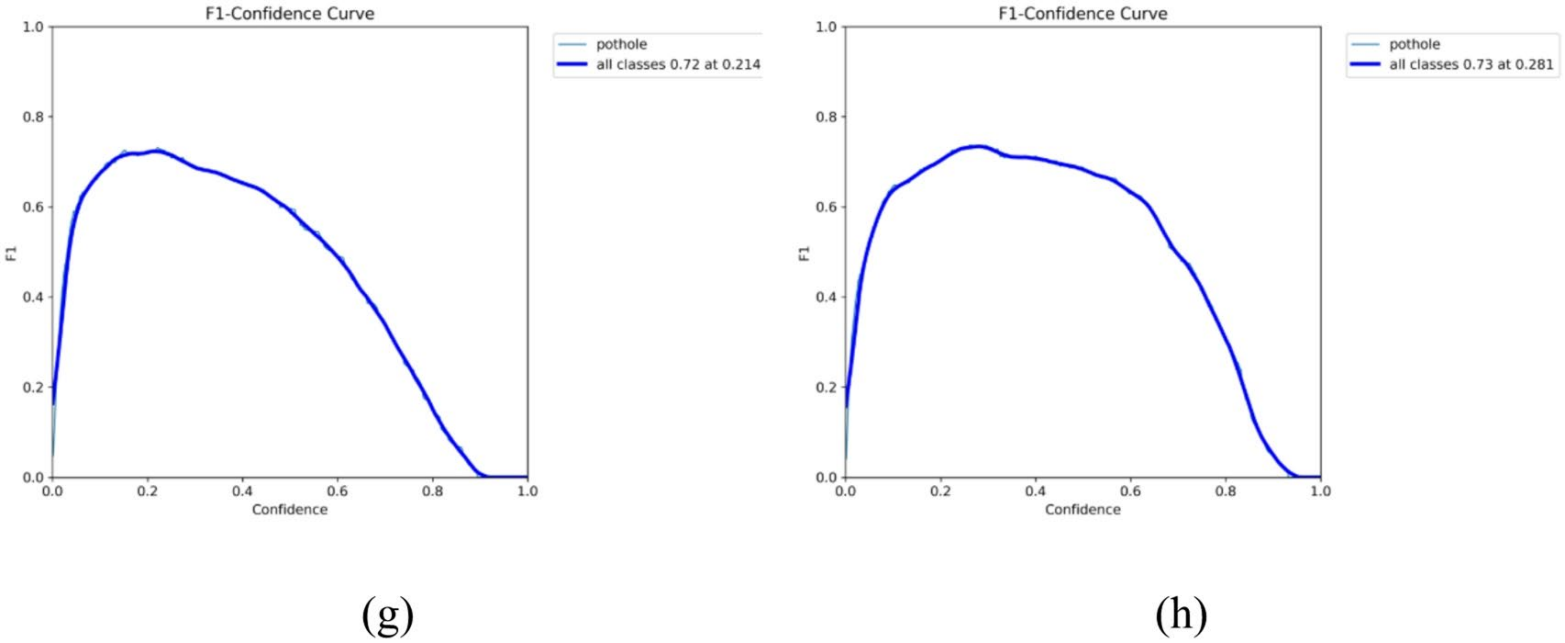
F1-Score Curves: (**a**) YOLOv5n; (**b**) YOLOv7-tiny; (**c**) YOLOv8n; (**d**) YOLOv10n; (**e**) YOLOv10s; (**f**) RT-DETR-r18; (**g**) YOLOv11n; (**h**) ECC-YOLO.

As illustrated in Fig. 10, the F1-Confidence curve evaluates the balance between precision and recall across different confidence thresholds for all compared models. Specifically, YOLOv5n achieves an F1 score of 0.69 at a low threshold of 0.244, showing stability suitable for recall-prioritized tasks. Conversely, while YOLOv7-tiny reaches a higher peak F1 of 0.72, its reliance on higher confidence levels suggests weaker fault tolerance. The YOLOv8 and YOLOv10 series (YOLOv8n, YOLOv10n, and YOLOv10s) demonstrate moderate peak F1 scores of 0.67, 0.61, and 0.60, respectively, though YOLOv10s exhibits a smoother curve indicative of better generalization potential. RT-DETR-r18 achieves a competitive F1 of 0.70, reflecting strong global modeling, but remains dependent on high-confidence predictions. The baseline YOLOv11n improves upon earlier YOLO versions by reaching a peak F1 of 0.72 with enhanced stability. Ultimately, the proposed ECC-YOLO outperforms all models, achieving the highest F1 score of 0.73 at a confidence threshold of 0.281. Furthermore, its curve is notably smoother with fewer fluctuations, demonstrating superior robustness, generalization capability, and well-rounded performance in multi-scenario pothole detection tasks.

**Fig. 11:**
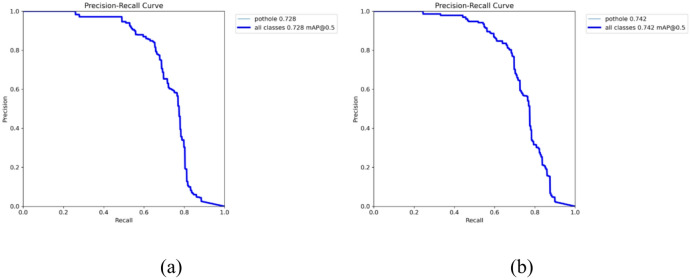
Precision-Confidence Curves: (**a**) YOLOv11n Algorithm; (**b**) ECC-YOLO Algorithm.

Figure 11 shows the comparison of Precision-Recall (P-R) curves between YOLOv11n and the proposed ECC-YOLO model in the pothole detection task. The mAP@0.5 of YOLOv11n is 0.728, while ECC-YOLO improves this to 0.742, marking an overall increase of 0.014. From the shape of the curves, it is evident that ECC-YOLO achieves significantly higher precision in the medium to high recall regions, indicating its ability to detect more targets while maintaining high recognition accuracy. This result validates the effectiveness of the introduced C3k2-DCNv2-Dynamic module, CDFA semantic fusion mechanism, and ELA-HSFPN feature enhancement structure in improving feature representation and detection performance. Compared to the baseline model, ECC-YOLO achieves better detection accuracy and stability without significantly increasing computational burden, making it well-suited for robust road pothole detection scenarios.

In object detection tasks, the spatial and scale distribution of training data plays a critical role in influencing the model’s learning performance. Particularly when targets exhibit noticeable small-size characteristics or positional concentration trends, the model may develop preferences or produce misjudgments in specific regions or scales. To better understand the data characteristics and their potential impact on model training, it is essential to visualize the center coordinates and size information of the target bounding boxes.

**Fig. 12:**
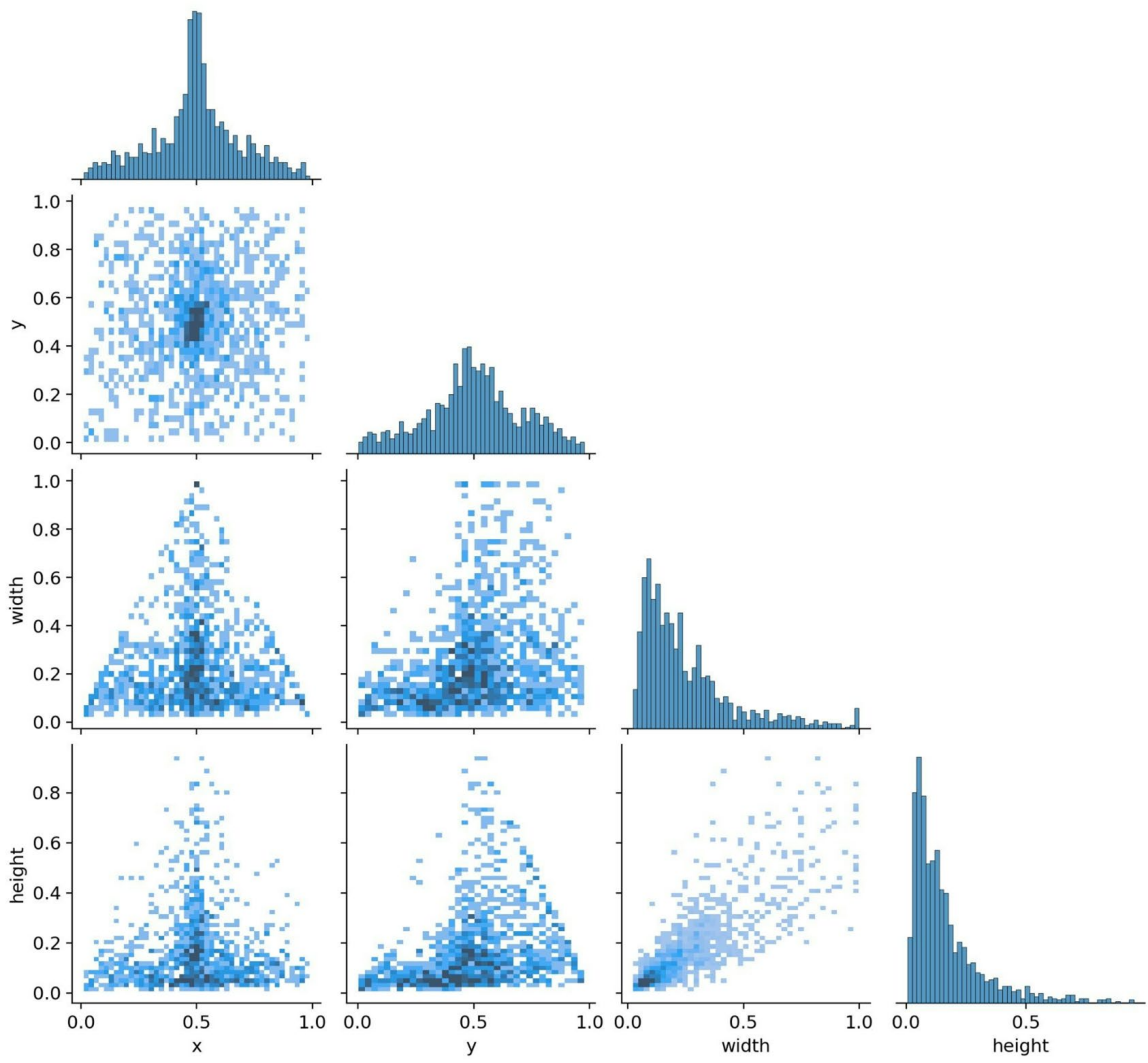
Pairwise distribution of normalized bounding box attributes.

To gain deeper insights into the spatial distribution and scale characteristics of the targets in the dataset, we plotted a pairwise distribution matrix of the normalized bounding box parameters—center coordinates (x, y) as well as width and height (see Fig. 12). The histograms along the diagonal show that the x and y coordinates are mostly concentrated around 0.5, indicating that target instances are primarily located near the center of the image. This spatial bias suggests that the model may benefit from incorporating a center-focused attention mechanism, such as adding center-prior weights in the loss function. In contrast, the distributions of width and height are clearly right-skewed, with most bounding box sizes being less than 0.3. This indicates a dominance of small-sized targets in the dataset, posing a greater challenge for detection tasks—particularly for models that rely on low-resolution feature maps. A moderate positive correlation is observed between width and height, suggesting that target dimensions tend to vary synchronously in both directions, although the relationship is not strictly linear. These findings can offer guidance for optimizing anchor box design or enhancing scale-aware mechanisms within the detector.

**Fig. 13:**
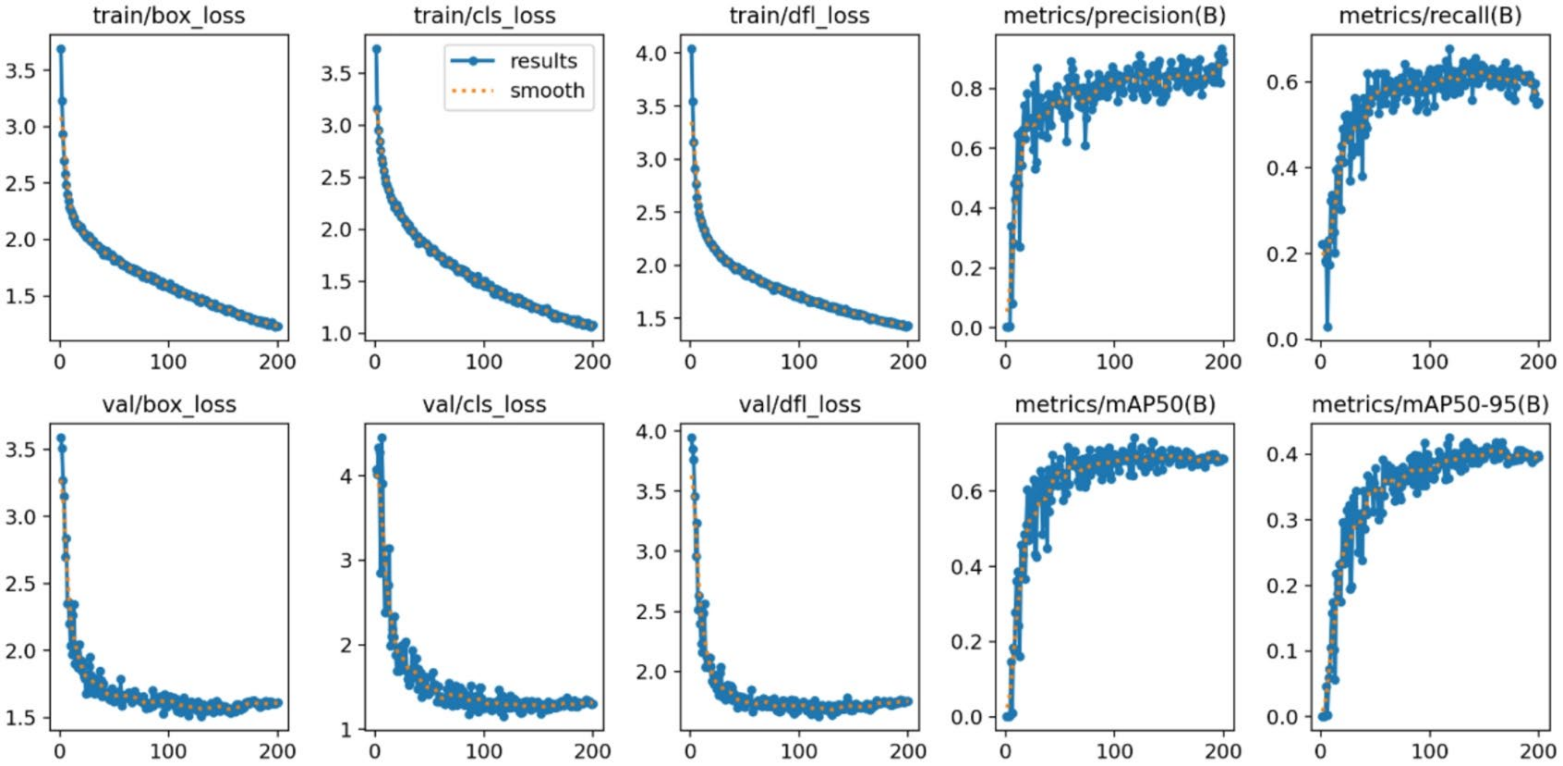
Loss and evaluation metric curves during training and validation of the ECC-YOLO Model.

To evaluate the model’s convergence during training and its generalization ability on the validation set, we plotted the loss function curves and evaluation metric trends for the ECC-YOLO model throughout the training and validation process, as shown in Fig. 13. The figure displays the changes in bounding box loss (box_loss), classification loss (cls_loss), and distribution focal loss (dfl_loss) during both the training and validation phases, along with key performance metrics on the validation set, including precision, recall, and mean Average Precision (mAP).

As shown in Fig. 13, all loss functions exhibit a steadily decreasing trend, with the training and validation loss curves closely aligned, indicating that no significant overfitting occurred during the training process. The classification loss and bounding box loss decrease rapidly within the first 50 epochs and then gradually stabilize, suggesting that the model quickly learned the basic features of the targets. The continuous decline of the distribution focal loss further indicates the model’s improving ability in refining bounding box localization. In terms of performance metrics, both precision and recall show a clear upward trend and become stable in the later stages of training, confirming the model’s strong target recognition capability. Both mAP@0.5 and mAP@0.5:0.95 continue to improve throughout the training process. Notably, mAP@0.5 reaches a high and stable value around the 150th epoch, demonstrating the model’s reliable detection performance across multiple scales and varying IoU thresholds. Overall, the ECC-YOLO model exhibits strong convergence and generalization ability during training.

### Ablation experiment

To verify the specific impact of the proposed modules (C3k2DCY, CDFA, and ELA-HSFPN) on overall performance, we conducted a series of ablation experiments based on the baseline YOLOv11n. By incrementally integrating these components and comparing Precision, Recall, and mAP@0.5, the contribution of each component is clearly identified, providing empirical evidence for the structural optimization.

**Table 4:** Ablation experiment results.

number	Experiment	Precision/%	Recall/%	mAP@0.5/%
1	YOLOv11n	82.8	65.4	72.8
2	YOLOv11n+C3k2DCY	80.5	63.5	72.3
3	YOLOv11n+CDFA	77.5	65.4	74.2
4	YOLOv11n+CDFA + ELA-HSFPN	81.2	64.4	73.2
5	YOLOv11n+C3k2DCY + ELA-HSFPN+CDFA	84.5	67.9	74.2

Table 4 details the performance impact of each configuration. A critical phenomenon observed is that introducing certain modules individually actually degrades specific metrics compared to the baseline. This behavior highlights the deeply interdependent nature of pothole detection challenges and the necessity of a coupled architectural design.

Specifically, when the C3k2DCY module is introduced alone, all three metrics decline (Precision drops to 80.5%, Recall to 63.5%, and mAP@0.5 to 72.3%). Because deformable convolutions fundamentally rely on sharp local feature gradients to learn accurate spatial offsets, deploying C3k2DCY in highly textured, noisy asphalt backgrounds without a prior noise-suppression mechanism causes its sampling points to drift chaotically, extracting corrupted geometric features. Conversely, adding only the CDFA module improves mAP@0.5 to 74.2% but decreases Precision to 77.5%. While CDFA actively amplifies boundary contrast to find hidden targets, doing so without the adaptive spatial geometric constraints provided by C3k2DCY inadvertently amplifies background artifacts, leading to an increase in false positives.

These isolated failure modes empirically prove that the full performance gain is only unlocked through their specific synergistic combination. The modules mutually resolve each other’s limitations: the CDFA module acts as an active background suppressor, mathematically providing the clean, high-contrast gradients required to prevent C3k2DCY from drifting. In return, the spatially adaptive C3k2DCY module precisely anchors onto irregular pothole boundaries, providing the strict geometric constraints needed to prevent CDFA’s amplified artifacts from triggering false positives. Finally, the ELA-HSFPN module guarantees that these accurately extracted, shape-adaptive, and contrast-enhanced local features are effectively propagated and fused across multiple scales. This progressive mechanism (background noise suppression, followed by shape-adaptive feature extraction, and concluding with edge-preserving multi-scale fusion) fundamentally explains the robust synergistic performance of the complete ECC-YOLO architecture.

### Quantitative error analysis and failure case discussion

While the proposed ECC-YOLO demonstrates significant overall performance improvements, a detailed quantitative error analysis is essential to fully understand the boundaries of its capabilities and its current limitations. Based on the normalized confusion matrix data (Fig. 9f) and subsequent error profiling, the residual prediction errors of ECC-YOLO can be quantitatively categorized into three main types: background false positives, false negatives or missed detections, and localization errors.

Analytically, the reduction in background false positives, from 39% in the baseline YOLOv11n to 30% in ECC-YOLO, quantitatively validates the effectiveness of the CDFA module in suppressing asphalt noise. However, an in-depth review of the remaining failure cases reveals specific extreme environmental conditions where the synergistic mechanisms of ECC-YOLO degrade.

First, water-filled potholes with high reflectivity constitute a major failure case. When a pothole is completely submerged in water, its surface acts as a mirror, reflecting the sky or surrounding vehicles. This optical phenomenon entirely disrupts the foreground-background gradient assumptions of the CDFA module, misleading the attention mechanism and resulting in missed detections or severe localization shifts. Second, in extreme low-light or nighttime scenarios, the visual contrast of the weathered asphalt degrades to near zero. Under such extreme conditions, the C3k2DCY module lacks the necessary edge gradients to learn valid spatial offsets, causing the deformable sampling points to fail in capturing the target’s geometric structure. Finally, for micro-potholes at extreme distances, the spatial resolution is inevitably compressed in the deeper layers of the network. Despite the edge-preserving efforts of the ELA-HSFPN module, the limited pixel representation of these tiny anomalies often leads to false negatives.

Discussing these failure cases provides crucial insights into the model’s limitations and highlights clear directions for future research, such as incorporating multi-modal data to overcome severe optical degradation in purely vision-based detection systems.

## Conclusion

This paper proposes an enhanced pothole defect detection algorithm based on the YOLOv11n architecture, ECC-YOLO, designed to overcome the challenges faced by traditional detection methods in complex road environments, such as uneven lighting, texture interference, and irregular shapes. To address these issues, the model first incorporates the C3k2DCY module to enhance its ability to model irregular pothole shapes and to better select key features, thereby enabling more precise feature representation. Secondly, the Contrast-Driven Feature Aggregation (CDFA) module is employed to increase the contrast between potholes and the background during feature fusion, highlighting edges and critical regions while effectively suppressing background noise and reducing false detections. Finally, the ELA-HSFPN module enables efficient multi-scale feature fusion by combining edge and semantic information, further improving the model’s localization and recognition capabilities. Experimental results demonstrate that the proposed method delivers excellent detection performance under complex conditions, exhibiting high robustness and strong generalization ability.

Despite the promising results, this study has certain limitations regarding the scale and diversity of the experimental dataset. Although extensive data augmentation techniques were employed to mitigate the constraints of the relatively small dataset (665 original images), simulated augmentations cannot fully encapsulate the extreme diversity of real-world road environments. For instance, scenarios involving severe weather conditions, nighttime illumination variations, and diverse asphalt materials across different geographical regions are not comprehensively represented. Furthermore, the current evaluation lacks cross-dataset validation, which leaves the model’s generalization capabilities to completely different road networks and external datasets partially unverified. Consequently, the model’s generalization capability may experience degradation when deployed in these unseen extreme conditions.

Future work will proceed along three main avenues to further enhance the model’s practical relevance. First, we will focus on constructing a larger-scale, multi-scenario dataset and conducting rigorous cross-dataset validation. This will allow us to proactively evaluate and improve the model’s generalizability across diverse geographical regions, completely different road conditions, and unseen environmental datasets. Second, to overcome the severe optical degradation observed in highly reflective, water-filled potholes, we plan to integrate multi-modal data fusion, combining vision with thermal or depth sensors. Most importantly, to fully capitalize on ECC-YOLO’s lightweight architecture, future efforts will prioritize practical deployment considerations. We intend to conduct hardware-specific optimizations, such as model quantization and TensorRT acceleration, to facilitate seamless integration into resource-constrained edge computing platforms.

## Data Availability

The dataset utilized in this study is publicly available on the Roboflow platform: https://public.roboflow.com/object-detection/pothole [27]. To ensure transparency and reproducibility, the complete source code, pre-trained models, and detailed implementation instructions for the proposed ECC-YOLO model will be made publicly available on GitHub immediately upon the acceptance of this manuscript.
